# Clinical Lectures on Diseases of the Eye

**Published:** 1863-12

**Authors:** E. L. Holmes

**Affiliations:** Chicago, Lecturer on Diseases of the Eye and Ear in Rush Medical College, and Surgeon of the Chicago Charitable Eye and Ear Infirmary


### ORIGINAL COMMUNICATIONS.

### CLINICAL LECTURES ON DISEASES OF THE EYE.

By E. L. HOLMES, M. D., of Chicago,

*Lecturer on Diseases of the Eye and Ear in Rush Medical College, and Surgeon of the Chicago Charitable Eye and Ear Infirmary.*

### *The Conjunctiva,—Anatomy, Physiology, and Pathology.*

GENTLEMEN:—We have before us to-day several cases, presenting different abnormal conditions of the Conjunctiva. I wish to urge upon you a careful study, not only of these cases but especially, of the whole subject of conjunctival inflammation. If not the most interesting, it is practically the most important part of our course, since, in the West, the number of cases of conjunctival inflammation and its sequelæ is far larger than the aggregate number of cases of all other diseases of the eye, which you will meet in practice.

I am convinced more patients become blind from the effects of these diseases than from all other causes. It would be difficult to estimate the physical distress endured for weeks and months by thousands of patients, afflicted with ordinary "sore eyes," in the Northwestern States. No one, except those brought in frequent contact with this class of patients, can imagine their mental sufferings in view of their own dismal prospects for the future, and of the sad condition of their families deprived of their support and care. Parents, thus suffering, are often forced to leave their children without moral and mental culture, and without restraint, and with evil habits such as idleness and ignorance always bring. Children in large numbers with diseases of the eye, neglected or improperly treated, have been left blind for life, or with so little vision as to be unfit for the ordinary duties of life.

Few diseases in the West are so universally considered of little significance, and yet become the cause of so much misery as those of which we are now speaking. The thought, that upon you will hereafter devolve the responsibility of treating such cases, will lead you, I hope, to study this subject faithfully. That you may the better understand the progress and treatment of these diseases, I shall first call your attention to a few points regarding the Anatomy, Physiology and abnormal conditions of the Conjunctiva.

If you dissect this membrane from the eye-lids and from the globe, you will have a circular piece of tissue, about two and a half inches in diameter, with an opening near the center, corresponding to the cornea. The Conjunctiva resembles, in its general structure, the other mucous membranes of the body. It is composed principally of fusiform and star-shaped cells and areola tissue. These cells are furnished with exceedingly minute tube-like appendices, which anastomose freely with those of other cells. The epithelium consists of a deeper layer of elongated cells, covered with a delicate layer of flat, hexagonal cells.

There are three distinct portions of this membrane, differing from each other not only in position, but in structure. The first portion, covering the cartilages of the lids, is composed of an epithelial layer and a firm areola tissue; it is provided with large numbers of papillæ, which give it under the microscope its peculiar velvety appearance. The second portion, the duplicature, comprises the loose folds, varying in extent in different individuals, which lie between the points where the Conjunctiva leaves the lids and joins the globe. It contains few papillæ but very large numbers of mucous glandulæ, somewhat resembling minute bunches of grapes. The semilunar fold at the inner angle of the eye is of the same structure as this portion of the Conjunctiva.

The third portion covers the anterior part of the sclerotica. It is transparent, less firm and thick than the palpebral portion, and has neither papillæ nor mucous glandulæ. Its epithelial layer extends over the cornea. The annular portion of the sclerotical conjunctiva, which encroaches upon the cornea, and which can be seen as a transparent ring at the circumference of the cornea, is called the limbus conjunctivæ. Between the sclerotica and conjunctiva is a thin layer of submucous areola tissue called the capsule of Tenon, the anatomy of which is of some little importance in operating for strabismus and in removing the eye.

The vessels which supply the Conjunctiva with blood are branches of the ophthalmic artery and the facial branches of the external carotid. The vessels of the sclerotic portion of the Conjunctiva are scarcely visible to the naked eye. The larger vessels, seen in this portion of the eye, belong really to the submucous tissue.

Branches of the fifth pair of nerves are very freely distributed to the palpebral portion of the Conjunctiva. The folds between the lids and globe possess comparatively little sensibility. The intimate relation of these nerves with the nerves regulating the motions, secretions and other functions of the eye, is shown by the manner in which irritation of the mucous membrane will produce a flow of tears, involuntary motions of the facial muscles, and closing of the lids.

The Conjunctiva undoubtedly furnishes the largest portion of the lubricating fluid, by which the movements of the lids and globe are facilitated. That the lachrymal gland contributes but a small part of this fluid may be shown by the fact, that the extirpation of the gland does not diminish the ordinary amount of moisture of the Conjunctiva.

In treating of conjunctivitis I shall at present confine my remarks principally to those forms, of it which you will most frequently meet in practice, viz.: the catarrhal, muco-purulent, purulent and scrofulous. But before we enter upon the study of these varieties of disease, it will be well to consider generally the progress and effects of inflammation in the Conjunctiva.

The inflammatory action, however produced, seems to commence first in the spindle and star-shaped cells, and in the deeper cells of the epithelium. These cells take on an increased activity, becoming enlarged and filled with granules and serum-like fluid ; as the process continues, the minute appendius of these cells also become enlarged and filled in the same manner. As a result of these changes there is an increased rapidity in the formation of new cells not only in the substance of the mucous membrane, but also in the epithelium. Accompanying this increased formation of cells, there is a congestion of the vessels and an infiltration of lymph into the tissues of the Conjunctiva, in consequence of which there is more or less swelling, which often extends to the whole substance of the lids. In proportion to the intensity of the inflammation there is a greater or less amount of secretion, composed of mucous and pus mingled with the other fluids of the eye. The relative quantity of pus and mucous depends upon the intensity, and possibly upon the character, of the inflammation, as in a case of mild muco-purulent conjunctivitis in which there may be scarcely a trace of pus in the discharge: or in a case of purulent inflammation in which the discharge is composed almost wholly of pus.

An inflammation of the Conjunctiva even in its severest forms may terminate in resolution and leave the eye in perfect health, or it may run a rapid course terminating in the destruction of important tissues, with vision to a greater or less extent impaired. It is curious to observe the differences in the effects of an inflammation in different cases, apparently analogous. As for instance, you may examine a neglected case of purulent conjunctivitis in an infant, in which you find sloughing of the cornea a week after the attack; in another case, apparently precisely similar, you may find the cornea uninjured, even though the disease may have existed nearly a month.

One of the most frequent unfavorable results of conjunctivitis is atrophy. The infiltration of lymph into the substance of the conjunctiva produces pressure upon the nerves, vessels, glandulæ, and disarranges the process of nutrition. Finally, as the products of inflammation become absorbed the Conjunctiva remains not only atrophied but also changed as regards the character of its tissues. Occasionally, as in the very remarkable case we have seen in the Clinic to-day, the Conjunctiva is not only so much atrophied as to pass direct from the edges of the lids to the globe, but is also transformed to a perfectly translucent, parchment-like membrane, covering the cornea. All the secretions of the eye are arrested, and the surface of the globe is perfectly dry. The finger rubbed upon the cornea produces no irritation. In other cases the atrophy occurs without so great transformation of structure. The functions may not be excessively deranged and yet the lids appear narrower and shorter than natural. In some cases the meibomian glands and the follicles of the cilia are destroyed; in others the roots of the cilia are displaced by the absorption of the surrounding tissues; in consequence of which, the cilia grow in an abnormal direction towards the globe, causing much discomfort to the patient, as in Trichiasis. Sometimes where there is great relaxation of the external tissues with contraction of the Conjunctiva, the whole lid is turned in, bringing all the cilia in contact with the globe. This condition is called Entropion.

Occasionally in this country—much more frequently in Europe I have reason to believe—there is formed, as a product of inflammation, a false membrane upon the surface of the Conjunctiva, especially of the lids. This exudation may be simply attached to the surface, or it may also be formed in the substance and upon the surface of the Conjunctiva as in the case of true diphtheritic conjunctivitis.

Before atrophy occurs, as just described, there is very often hypertrophy of the Conjunctiva, especially of the folds, lying between the lids and the globe, and of the plica-semilunaris. While this condition exists, the Conjunctiva, except upon the globe, is covered with elevations, composed of cells and a jelly-like exudation. This condition is termed Trachoma—or in common language, "granulated lids." On the lids these "granulations," according to some writers, are simply hypertrophied papilla; at the duplicature of the Conjunctiva, they are composed principally of jelly-like granules.

The processes, as above described, are liable to be influenced by other diseases of the system, as is often observed in cases of scrofulous diathesis, of small pox, measles and other eruptive diseases. Phlyctenular conjunctivitis—Herpes conjunctivæ—is seldom found except in patients suffering more or less from strumous diathesis. The small elevations upon the Conjunctiva peculiar to these complications, seem to be produced in nearly the same manner as the "granulations" above described. The inflammation appears at first to be confined to small circumscribed points, where by the increased formation of cells and exudation of lymph, small elevations are formed. These elevated points often ulcerate, leaving an irritable surface, from which the inflammation extends to other parts of the Conjunctiva and cornea, with the same results as in other forms of conjunctivitis.

These few explanatory remarks will enable you, I trust, to understand even by simple inspection, what processes have produced the abnormal changes, you will observe in very many cases to be brought before you this winter.

